# Accessibility calculation and equality evaluation of medical facilities for COVID-19 pandemic treatment: A case study of the Wuhan metropolitan development zone

**DOI:** 10.1371/journal.pone.0272458

**Published:** 2022-08-02

**Authors:** Xiumei Shen

**Affiliations:** School of Architecture, Southeast University, Nanjing, Jiangsu, China; China University of Geosciences, CHINA

## Abstract

Medical facility equality is a critical metric for determining equal access to medical care. Their spatial distribution is important for effective pandemic treatment and daily prevention in cities. This paper used the Kernel Density Two-Step Floating Catchment Area (KD2SFCA) and shortest distance methods to calculate the accessibility of designated COVID-19 Fangcang hospitals and fever clinics in the Wuhan Metropolitan Development Zone. Their equality was evaluated by the Gini coefficient and Lorentz curve. Several results were obtained: (1) The facilities’ accessibility declines radial from the central to peripheral areas. (2) Most of the demand points in the study area can reach the medical facilities for COVID-19 pandemic treatment within 60 minutes. (3) For the spatial distribution of these facilities, the equality evaluated for different time thresholds differed significantly, with long time thresholds having better equality than for short time thresholds. (4) While the distances distribution of fever clinics is balanced, the equality gap in various areas remains enormous when considering population distribution. Suggestions for optimizing the spatial distribution of pandemic treatment medical facilities in Wuhan are proposed, and which will serve as references for the planning of Wuhan’s pandemic medical facilities in the future.

## 1. Introduction

Coronavirus disease 2019 (COVID-19) initially appeared in 2019 and quicky spread over the world [1, 2]. The occurrence of epidemics has had a significant impact on the global natural environment and socioeconomic activities [3], and the sudden increase in infections has led to a shortage of medical resources in many countries and regions [4, 5]. Wuhan used to be the city with the worst COVID-19 outbreak in China. Following the outbreak, the Wuhan Municipal Health Commission announced the list of 61 fever clinics on January 20, 2020 for the screening and diagnosis of potential COVID-19 patients. At the same time, the list of designated COVID-19 hospitals was released in five batches; these 48 hospitals were especially dedicated for the treatment of severe and critical patients. However, due to the large number of COVID-19 patients, the number of beds in those hospitals remained insufficient. On February 5, 2020, Wuhan began opening Fangcang shelter hospitals (hereafter referred to as "Fangcang hospitals"), with a total of 15 Fangcang hospitals in operation to treat mild and moderate patients. The first Fangcang hospital was built in Wuhan to combat the spread of COVID-19 and give patients with timely basic medical care. The operation of them have significantly increased the accessibility of medical services for COVID-19 treatment in a short time. COVID-19 has been effectively controlled in China since March 2020. However, the allocation of medical facilities for pandemic treatment can be further improved in order to prevent future pandemics.

Equality in the allocation of medical and health resources is one of the key aims pursued by health policy makers and health systems; it is also one of the basic prerequisites for the sustainable development of medical and health services [6]. Especially after the outbreak, the supply and demand became drastically uneven. Thus, evaluating the equal distribution of medical facilities can assist policymakers in better allocating limited resources and optimizing the spatial layout of medical facilities in the future plan.

The majority of current research on the equality of residents’ access to medical services focuses on two aspects. On the one hand, some scholars analyze accessibility from the demand side’s demographic attributes (e.g., age, gender, race, income, etc.) [7–9]. On the other hand, some scholars are concerned with the accessibility and spatial distribution of facilities. For the latter, scholars quantify the disparities or degrees of accessibility of medical facilities in a research area (including the number of beds, doctors, and registered nurses) and then use the Gini coefficient, Theil index, or multi-norm framework to evaluate inequality between places [10–12]. Since the outbreak of the COVID-19 pandemic, scholars have been very concerned about the distribution of medical resources. By analyzing the accessibility of medical facilities in a given area, they were able to identify places and populations where medical resources were in short supply, providing a basis for urban policy makers to respond to pandemics in the future. These studies included analyzing the accessibility of ICU beds in Florida during the COVID-19 pandemic using 3SFCA [13], calculating the accessibility of COVID-19 healthcare resources in the 20 largest Brazilian cities using a balanced float catchment area approach [14], and measuring the spatial accessibility of COVID-19 healthcare resources in Illinois using an enhanced two-step floating catchment area (E2SFCA) [15].

Accessibility is a decisive factor that affects the level of medical service equalization [16]. And it is also a popular method for evaluating the distribution of medical facilities and finding service gaps [17]. Distance-based models, the Huff model, the potential model and its improved versions [18–21], and the Two-Step Floating Catchment Area (2SFCA) algorithm and its enhancement [22–26] have all been proposed in the literature in recent years for calculating the accessibility of public facilities. Both the potential model and the 2SFCA method have been widely used and developed, as they take into account both supply and demand scale components, as well as the distance cost [22].

Despite the abundance of research findings on medical facilities accessibility and the diversify of research methods used, several issues remain unresolved. First, most prior studies identified residential area or village as points of interest (POI) for medical services demand [10, 11]. In fact, there is a demand for medical services in buildings that are either residential or non-residential. Moreover, the likelihood of an outbreak is greater in public facilities, such as vegetable markets and commercial buildings. Second, the research is insufficiently accurate. Many studies consider street administrative districts to be the smallest unit of demand points [17, 27]. However, due to historical reasons, the population distributions in many streets are not balanced, and the population densities of different communities vary substantially in many large cities. If these considerations are ignored, the study’s findings will be far from reality. Third, travel time and distance are too idealized. As early as 2011, Wang highlighted the benefits of combining network map API (Application Programming Interface) data with ArcGIS to analyze accessibility [28]. However, many studies still use ArcGIS road network datasets to calculate travel time by setting the driving speed [23, 27], while others employ a location–allocation model (LA model) to calculate the distance between demand points to hospitals [11, 12]. Due to real-time traffic congestion and traffic light conditions, the actual time taken from demand points to hospitals will fluctuate significantly from the model’s estimations.

The current paper uses population data with a 1 km^2^ accuracy to solve the problem mentioned above, and the demand point is the centroid of each km^2^ plot. The demand points of diverse land properties are therefore covered, and the research accuracy is improved. This paper uses real-time data from Amap’s network map API service (https://lbs.amap.com/api/javascript-api/example/map/map-english) to acquire more accurate journey time and distance. An growing number of studies have used network map navigation data in recent years, demonstrating the validity of this method [10, 29, 30].

In general, the current paper gathered data from the Wuhan Municipal Health Commission on the number of beds in each designated and Fangcang hospital during the COVID-19 pandemic in early 2020. The accessibility and equality of the distribution of these facilities in different time thresholds were analyzed using the network map API service of Amap and Python script language, based on the geographic information system (GIS) and Gini coefficient. The specific research objectives were as follows: (1) calculate the accessibility of designated hospitals in 10, 30, and 60 minutes and evaluate their equal distribution; (2) calculate the accessibility of Fangcang hospitals and fever clinics in 15, 30, and 60 minutes and evaluate equal distribution; (3) propose suggestions for optimizing the spatial layout of these facilities based on the above evaluation results.

## 2. Study area and data sources

### 2.1 Study area

**Fig 1:**
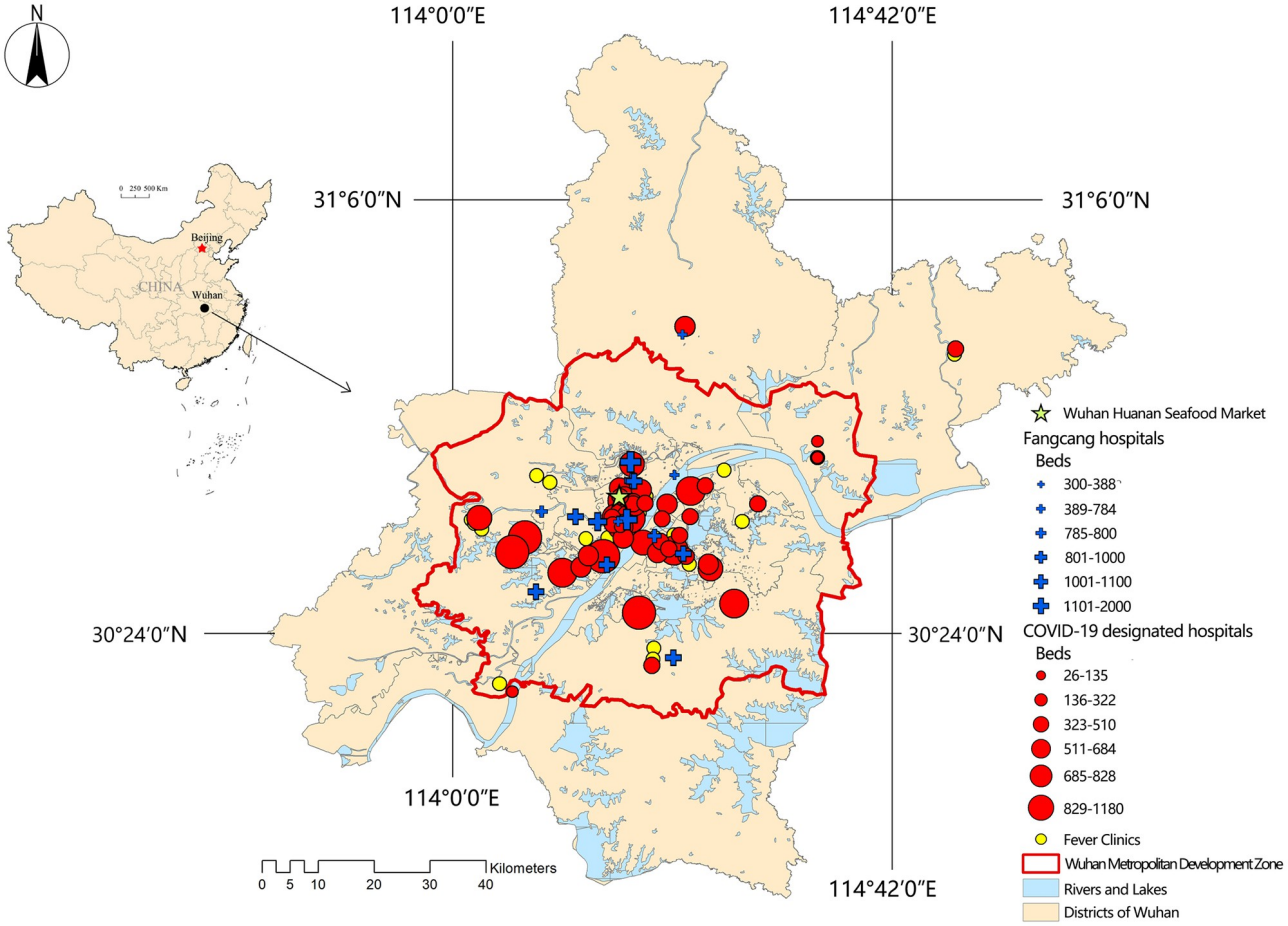
Distribution of medical facilities for pandemic treatment in the Wuhan MDZ.

**Fig 2:**
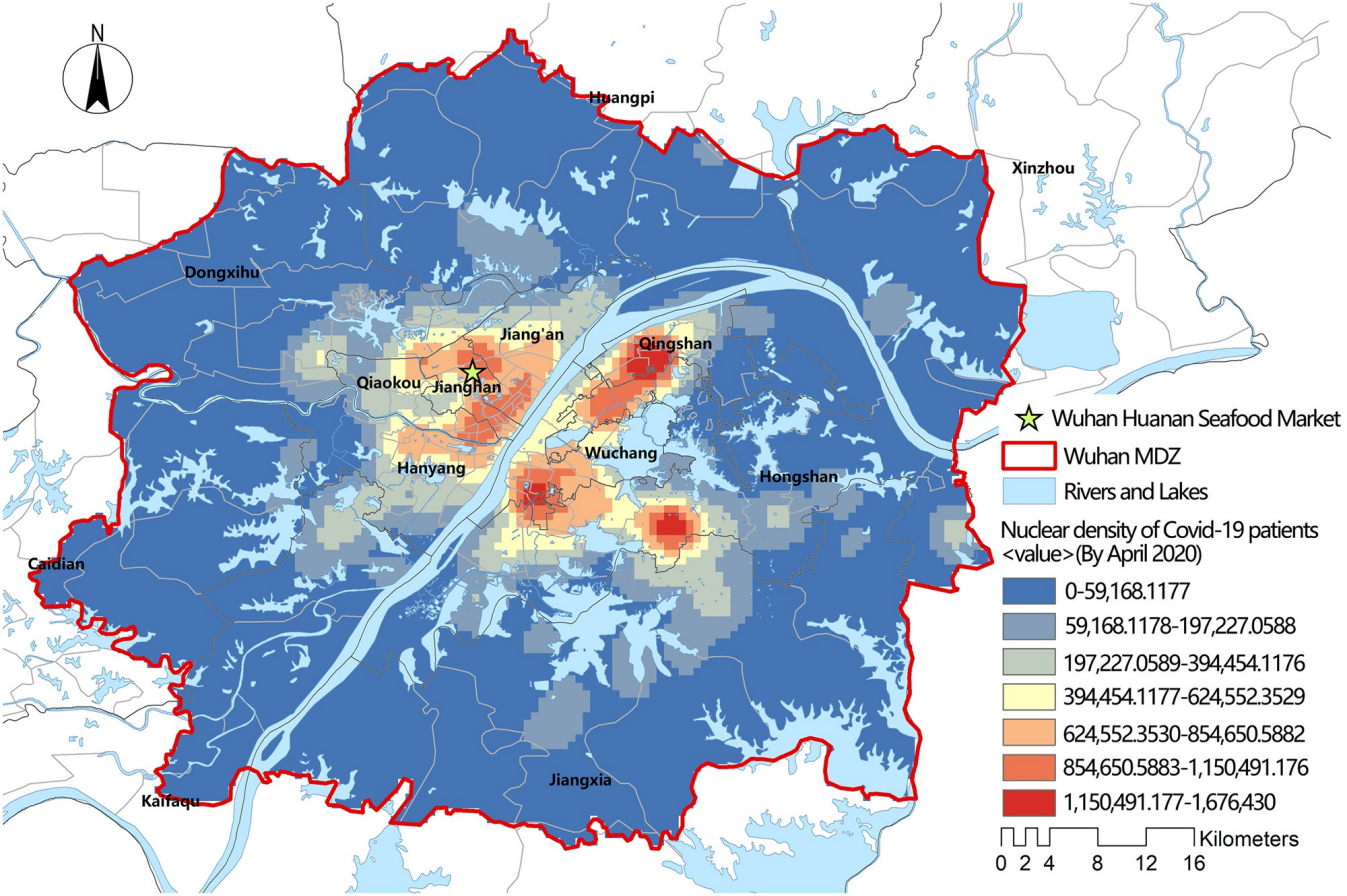
Nuclear density estimation map of COVID-19 patients in Wuhan city (April 2020).

Wuhan is the capital city of Hubei Province. About 84.5% of permanent residents in Wuhan are concentrated in the Wuhan Metropolitan Development Zone (MDZ, the geographical division is shown in Fig 1). In 2020, the communities with the most severe COVID-19 epidemic cases were clustered in this area (Fig 2), and the first set of patient discoveries in Wuhan occurred at the Huanan Seafood Market. This research focused on the Wuhan MDZ in order to improve the research’s relevance.

**Fig 3:**
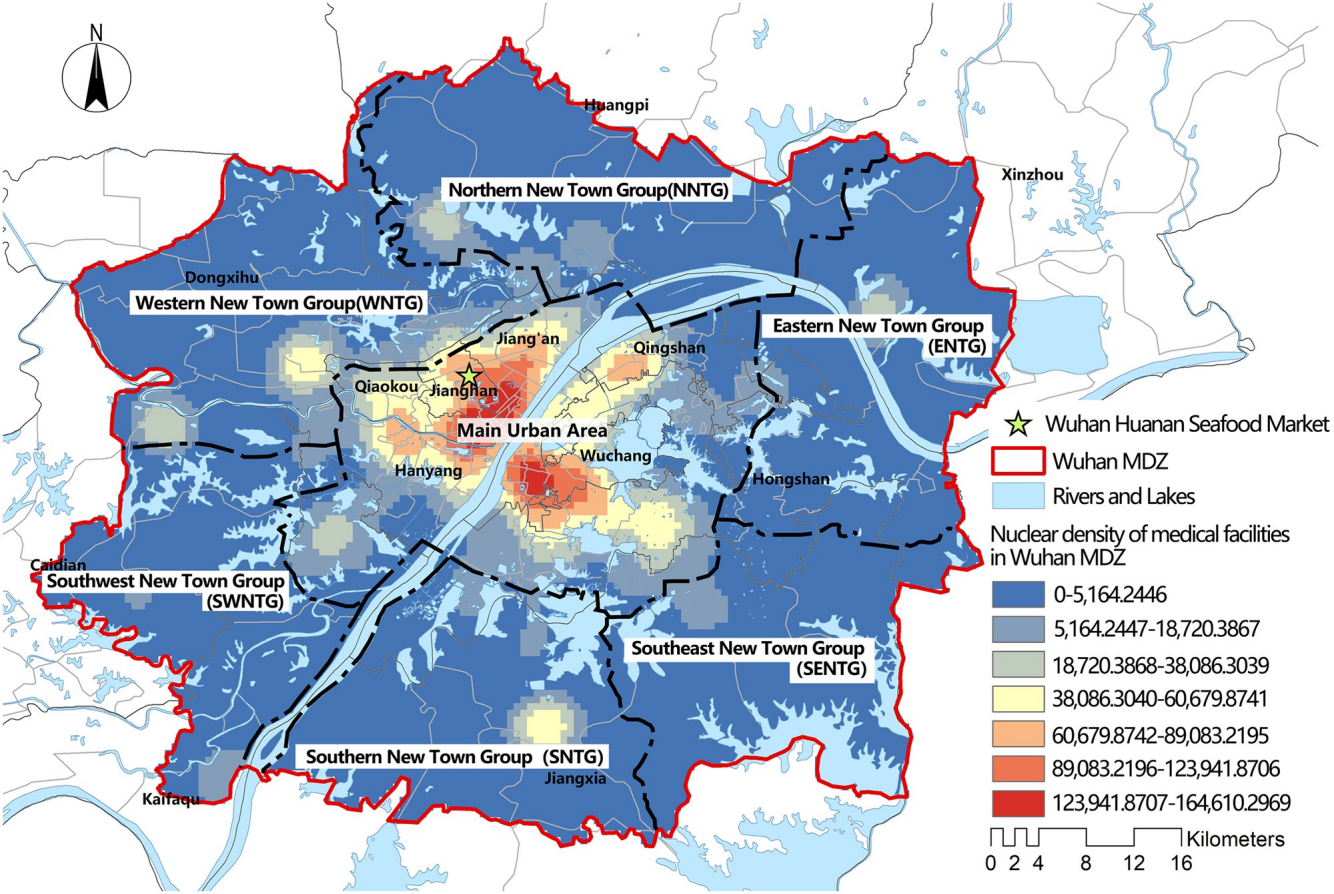
"1 + 6" space structure and nuclear density estimation map of medical facilities in the Wuhan MDZ.

According to the Wuhan urban master plan (2010–2020), the Wuhan MDZ includes the main urban area of Wuhan and the six New Town Groups that are of a "1 + 6" space structure (Fig 3). The six new town groups are the Eastern New Town Group, Southeast New Town Group, Southern New Town Group, Southwest New Town Group, Western New Town Group, and Northern New Town group (in the following pictures, they are referred to as ENTG, SENTG, SNTG, SWNTG, WNTG, and NNTG, respectively). The Wuhan Health Yearbook (2019) states that there were 4639 health institutions in the city by the end of 2018 (including 398 hospitals and 17 disease control institutions). Fig 3 shows the kernel density estimates from the ArcGIS kernel density analysis tool for all medical facilities within the Wuhan MDZ.

In the early stages of the epidemic in Wuhan (particularly before 20 February 2020), there was a significant disparity between patient demand for medical care and medical resource rationing. Despite the fact that the designated hospitals’ service regions included the majority of the main urban area, 31.2%-62.5% of patients’ actual distance and time to travel exceeded their wishes [31]. Figs 2 and 3 reveal a mismatch between the spatial distribution hotspots of COVID-19 patients and the spatial distribution hotspots of medical facilities in Wuhan at the time. Overall, the layout of pandemic treatment facilities in Wuhan does not correspond to the needs of patients, and some New Town Groups have medical service blind spots. Wuhan has achieved a stage success in the "battle against the epidemic" with the cooperation of all parties, but the struggle against the epidemic is a long one that will require additional optimization of medical resource allocation to halt the pandemic.

### 2.2 Data sources and processing

#### 2.2.1 Data on medical facilities for COVID-19 pandemic treatment

The lists of designated COVID-19 hospitals, Fangcang hospitals, and fever clinics as well as the number of open beds were gathered from data provided on the Wuhan Municipal Health Commission’s official website from January 20 to February 24, 2020. There were 61 fever clinics, 48 designated hospitals, and 15 Fangcang hospitals. Fig 1 depicts the general spatial distribution. The coordinate points of these medical facilities on Amap were obtained using Python script.

#### 2.2.2 Data on population

**Fig 4:**
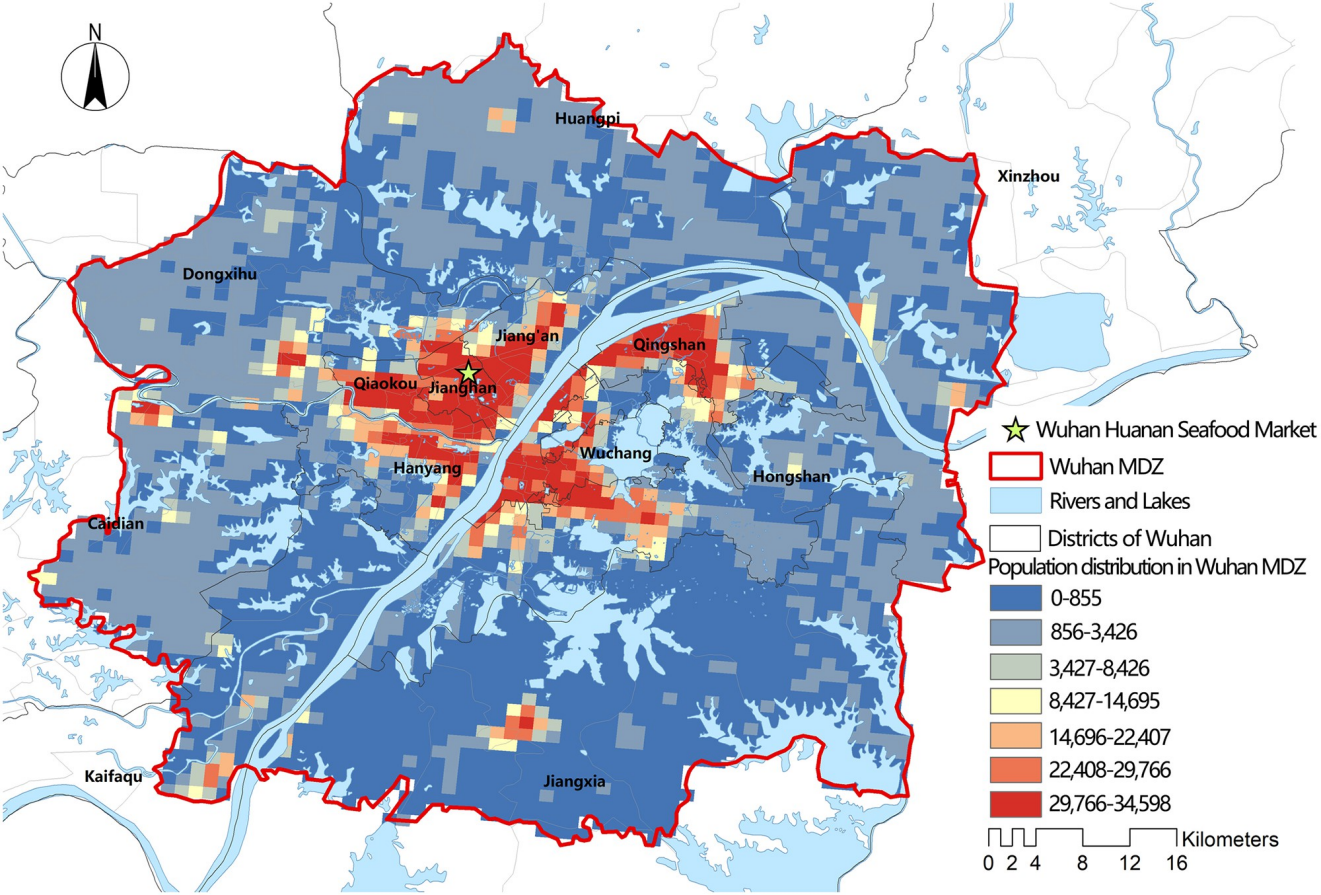
Population distribution of the Wuhan MDZ for every km^2^.

The population data came from China’s National Bureau of Statistics’ Sixth Population Census Data. The data was imported into ArcGIS 10.6 and converted into 1 km X1 km grid data using mathematical models. The mathematical model was first proposed by researchers from the College of Earth Science and Engineering of Hohai University, who aimed to establish a model by using the correlation between Tencent location data and demographic statistics data for a specific administrative zone. The rationality of this model was verified by detailed data [32]. The positioning method of Tencent location data is A-GPS hybrid positioning, which is based on the combination of GPS and GSM networks. About 90% of the positioning request had an accuracy of less than 22.5 m (https://heat.qq.com/production.php accessed on May 2019). Fig 4 shows how such data is more precise than a research based on district streets. Using the centroid of the population per km^2^ as the demand point, we can see that the resulting points include not only residential buildings, but also other types of buildings.

#### 2.2.3 Data on travel distance and time

**Fig 5:**
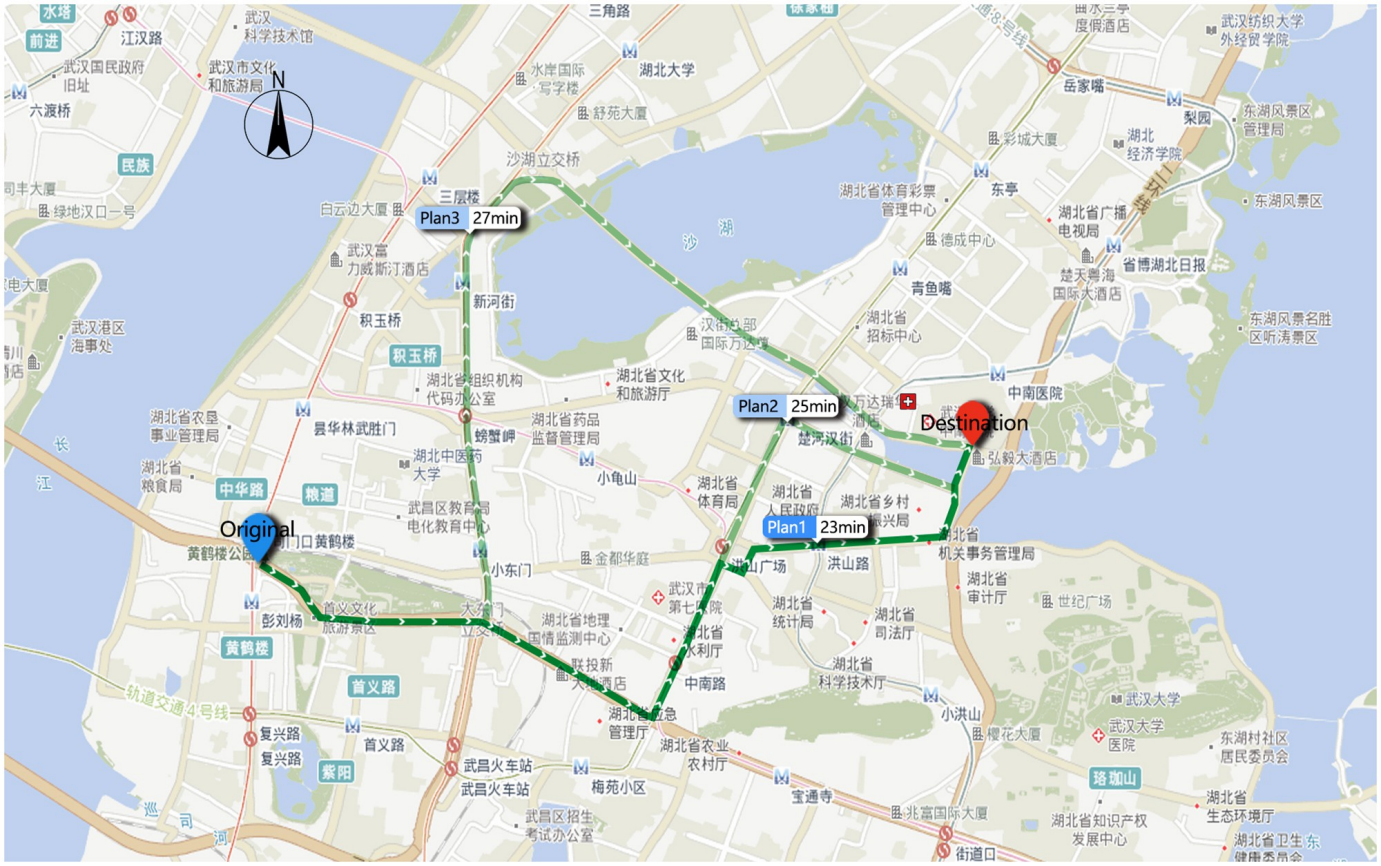
Navigation diagram of selected origin–destination (OD) routes.

Travel distance and time were obtained using the network map API service of Amap and Python script language, based on the location coordinates of demand points and medical facilities. The travel time of each time period is varies due to the influence of real-time traffic conditions. In order to reduce the amount of data, we selected off-peak hours on weekdays to obtain the travel times and their corresponding road distances (i.e., 9:00–11:00 a.m. on August 4, 2020). The off-peak period was chosen because, during a pandemic, people’s travel time will be shortened because traffic congestion will be lessened to some extent. The following was the procedure: First, ArcGIS 10.6 was used to extract the centroid of the population per km^2^ in the study area as the demand point for medical needs. Second, the POIs of pandemic treatment facilities were requested from Amap in batches, and the POIs were set as the destinations. The third step was to calculate the travel times and distances. A demand point and a destination were chosen to demonstrate these processes, as shown in Fig 5. Residents who need medical treatment during a pandemic will be unable to use public transportation and will have to drive; thus, the travel mode has been set to driving. The demand point and destination coordinates were then entered into Amap, which normally proposes 1–3 routes when the navigation function is called. Generally speaking, route one takes the shortest time, and most people take the quickest path. Therefore, route one was chosen as the study’s data source. Then, the best travel route and time from all demand points to facilities would be generated. The travel distance and time data for the suggested routes are shown in Table 1.

**Table 1:** Travel distances and times of different routes.

Routine ID	Demand point ID	Hospital ID	Distance (m)	Time (min)	Traffic mode
1	D1	H1	36009	41	Driving
2	D2	H1	29110	44	Driving
3	D3	H1	27636	41	Driving
4	D4	H1	27263	47	Driving
…	…	…	…	…	…
310482	D3303	H94	45279	53	Driving

## 3. Methodology

### 3.1 Accessibility calculation

The number of beds in designated and Fangcang hospitals is an essential aspect in their medical treatment capabilities; however, although they are unrelated to the treatment capabilities of fever clinics. As a result, this paper used two methods to calculate the accessibilities of these facilities.

#### 3.1.1 KD2SFCA methods

The 2SFCA algorithm was first proposed by Radke and Mu in 2000 [24]. This was further improved by Luo and Wang [33] who named their version as 2SFCA. Various varieties of 2SFCA have been proposed as a result of more research and growth by scholars, including the extension of distance attenuation model [34, 35], the extension of search radius [36, 37], the expansion of competition between demand and supply [38–40], and expansion based on travel mode [41, 42].

The current paper used the kernel density 2SFCA (KD2SFCA) to calculate accessibility of Wuhan COVID-19 designated hospitals and Fangcang hospitals. KD2SFCA was proposed by Dai et al. [43], and is an extension of 2SFCA, which is similar to E2SFCA [44] in that it incorporates a distance decay function. Within the search radius, E2SFCA segments the distance decay function by distance, whereas KD2SFCA adds a kernel density function-type distance decay function.

The supply–demand ratio at each demand point within a given time frame is represented by the KD2SFCA values. The calculation was split into two steps, and the mathematical model is shown below.

In the first step, for every hospital (j):

$${\text{D}}_{\text{j}}=\frac{{\text{S}}_{\text{j}}}{\sum_{\text{k}\in \left[{\text{d}}_{\text{kj}}\leq {\text{d}}_{0}\right]}{\;\;\;\;\text{P}}_{\text{k}}f\left({\text{d}}_{\text{kj}}\right)}$$ (1)

In the second step, for every demand point (i):

$${\text{A}}_{\text{i}}=\sum_{\text{j}\in \left[{\text{d}}_{\text{ij}}\leq {\text{d}}_{0}\right]}{\;\;\;\;\;\text{D}}_{\text{j}}\;f\left({\text{d}}_{\text{ij}}\right)$$ (2)

In the first step, based on the location j of a hospital, all demand points k were searched within a given travel time coverage from hospital j, then calculated the supply–demand ratio D_j_ in this range. In formula (1), d_kj_ is the travel time between hospital and demand point, d_0_ is the travel time threshold of the hospital’s service coverage, P_k_ is the demand within this service coverage (represented by the number of permanent residents in this paper), and S_j_ denotes the hospital’s supply capacity, which was represented by the number of beds in this research.

The second step was to search for all hospitals j within a given travel time (d_0_) threshold for each demand point i. Then summed up the supply–demand ratios of these hospitals to calculated the accessibility of each demand point A_i_.

People tend to prioritize the hospital that is closer to them within the same service coverage; therefore, the distance attenuation function was introduced here, and its expression is shown in formula (3):

$$f\left(\text{d}\right)=\left\{\begin{array}{l} e^{-d^{2}/k},d\leq d_{0}\\ 0,d>d_{0} \end{array}\right.$$ (3)

The longer the distance and time, the less probable it is that people will choose this hospital for medical treatment. As a result, the mathematical formula for KD2SFCA can be further integrated as follows:

$${\text{A}}_{\text{i}}\sum_{\text{j}\in \left[{\text{d}}_{\text{ij}}\leq {\text{d}}_{0}\right]}\frac{{\text{S}}_{\text{j}}\;f\left({\text{d}}_{ij}\right)}{\sum_{k\in \left[d_{kj}\leq d_{0}\right]}P_{k}\;f\left(d_{kj}\right)}$$ (4)

#### 3.1.2 Shortest distance

Fever clinics are dedicated clinics for screening suspected infected patients during the pandemic. As they do not require in-patient beds and the medical technology necessary is more common, we hypothesized that the supply capacity of each fever clinic was basically the same and that the main variation between them was their distance from the demand point. Therefore, the shortest distance was used to measure the accessibility of fever clinics in this research. Because it is critical for patients to be diagnosed and treated as soon as possible during the pandemic, the shortest distance in this research related to the cost of the shortest path when the Amap navigation system was called.

The shortest distance is a prominent metric that measures medical facilities’ accessibility. It is a very clear concept that is easy to communicate to policy makers and can be easily understood by public health researchers with a basic knowledge of GIS [11]. It can identify the distance between demand points and hospitals, the longer the distance, the lower the accessibility [16].

#### 3.1.3 Threshold of travel time

There are various methods for determining the travel time threshold based on the kind of facilities, service capacity of facilities, density of population, and mode of transportation [36, 45, 46]. Usually, the larger the scale and the better the service capacity of the facilities, the more attractive the demand point is, and the longer travel time people can accept. Since it is difficult to find an in-patient bed during the COVID-19 pandemic, the acceptable travel time can be extended infinitely. However, as severe patients must be treated as soon as possible in designated hospitals, the concepts of "Platinum Ten" and "Golden Hour" were adopted. The "Platinum Ten" is a concept that all types of emergency rescue and ambulance teams are familiar with. It is the time frame in which emergency crews assess the situation and begin treating and transporting patients once they get on the scene. The “Golden Hour,” which has been used in rescue for more than two decades, refers to the time when sophisticated medical treatment is required to increase one’s chances of survival [23, 47]. In addition, 30 mins is a usual time threshold setting for researches, which is in line with previous studies [8].

Considering that Fangcang hospitals mostly treat mild to moderate COVID-19 patients and the demands of patients in fever clinics are not so urgent, the minimum travel time was set at 15 mins. The other time limits were 30 and 60 mins, which were the same as those of designated hospitals.

### 3.2 Gini coefficient and Lorentz curve

The Gini coefficient and Lorentz curve were used to evaluate the equality of accessibility distribution of COVID-19 medical facilities over time in this study. Initially, the Gini coefficient was employed to assess the income gap between residents of a country or region, but it has increasingly been utilized to evaluate the equality or inequality of the spatial distribution of public service facilities [6, 10, 48]. It is a measure of equality that ignore the spatial distribution of data [49]. Meanwhile, the Lorentz curve ranks the accessibility values of different regions from small to large, then depicts the cumulative ratios of demand points and accessibility.

The Gini coefficient represents the inequality of accessibility distribution. Theoretically, if medical facilities are equally accessible in each demand point, the Gini coefficient is 0, indicating absolute equality. The Gini coefficient G formula is as follows:

$$\text{G}=1-\frac{1}{\text{m}}\sum_{\text{i}=1}^{\text{m}}\frac{\left({\text{C}}_{\text{i}}+{\text{C}}_{\text{i-}1}\right)}{\sum {}_{\text{i}=1}^{\text{m}}{\text{C}}_{\text{i}}}$$ (5)

where m is the total number of demand points, and C_i_ is the ranking cumulative value of demand point accessibility. The calculation process is as follows: rank and accumulate the accessibility A_i_ of m demand points from little to large, then sum of A_1_ to A_i_ to get C_i_.

## 4. Results

### 4.1 Accessibilities of different types of medical facilities for pandemic treatment

**Fig 6:**
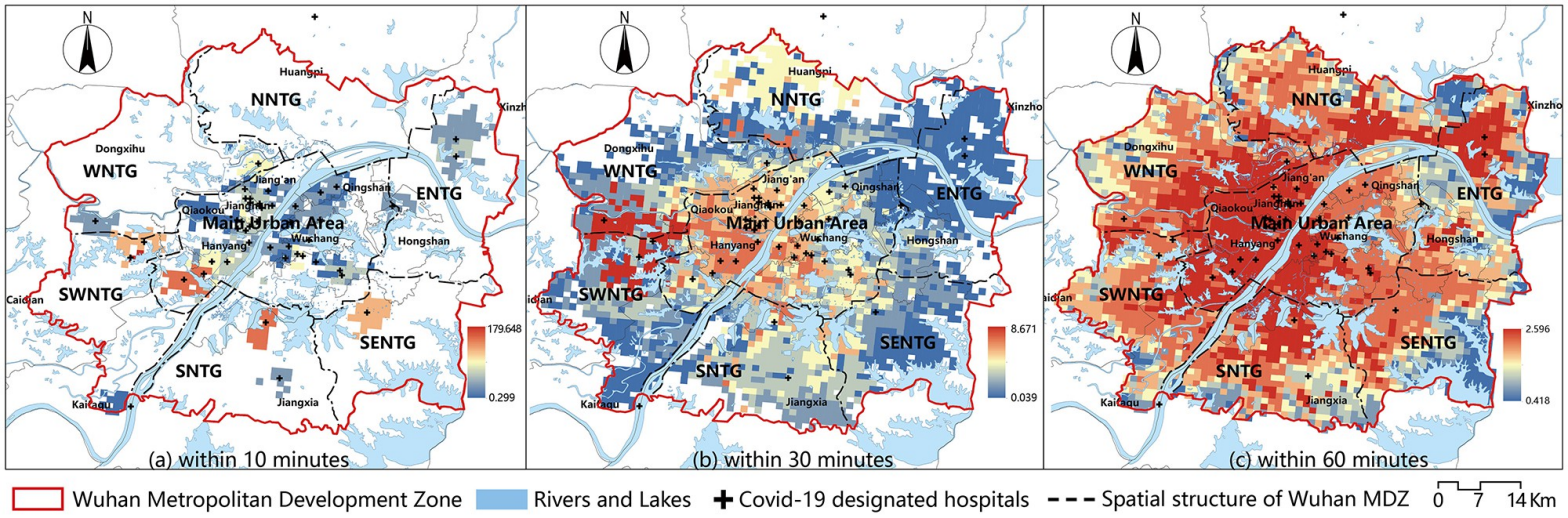
Accessibility of designated hospitals. (a)Within 10 minutes, (b) Within 30 minutes, (c) Within 60 minutes.

Using the designated hospitals as starting points, we selected the demand points with travel times of 10, 30, and 60 mins, respectively, and calculated their accessibilities using Formula 4. The results were then visualized in ArcGIS 10.6 using the Natural Breaks method (Jenks). As shown in Fig 6, the redder the color, the greater the accessibility, and the bluer the color, the worse the accessibility. According to the findings, there were 451 demand points in the Wuhan MDZ that could reach designated hospitals within 10 mins, accounting for 13.65% of the total. The main urban area was where these demand points were concentrated. The areas with the best accessibility were near the Leishenshan and Huoshenshan hospitals, as well as the West Hospital of the Wuhan Union Hospital of China. Despite the fact that the main urban area has more designated hospitals, accessibility within the 10-min limit was generally low (Fig 6a). In terms of spatial distribution, designated hospitals’ 30 mins service area could span a wide area, and 75.80% of demand points could reach designated hospitals in 30 mins. The northern area of the Southwest New Town Group and the southern area of the Western New Town Group had the best access within 30 mins. The main urban area’s accessibility was generally satisfactory. The Eastern New Town Group, on the other hand, has the worst accessibility. The accessibility value in the main urban area gradually decreased from southwest to northeast. Fig 4 show that in the northeast of the main urban area, the degree of coupling between accessibility and population distribution was low; thus, this area’s accessibility needs to be improved (Fig 6b). Furthermore, we discovered that all demand points in the Wuhan MDZ could reach designated hospitals within 60 mins (Fig 6c) and that the majority of areas in the main urban area were accessible. Accessibility in the northeast was slightly worse than in other areas similar to the results of the 30 min. The Southeast New Town Group accounting for the biggest proportion of the places with inadequate accessibility on the eastern edge of the Wuhan MDZ.

**Fig 7:**
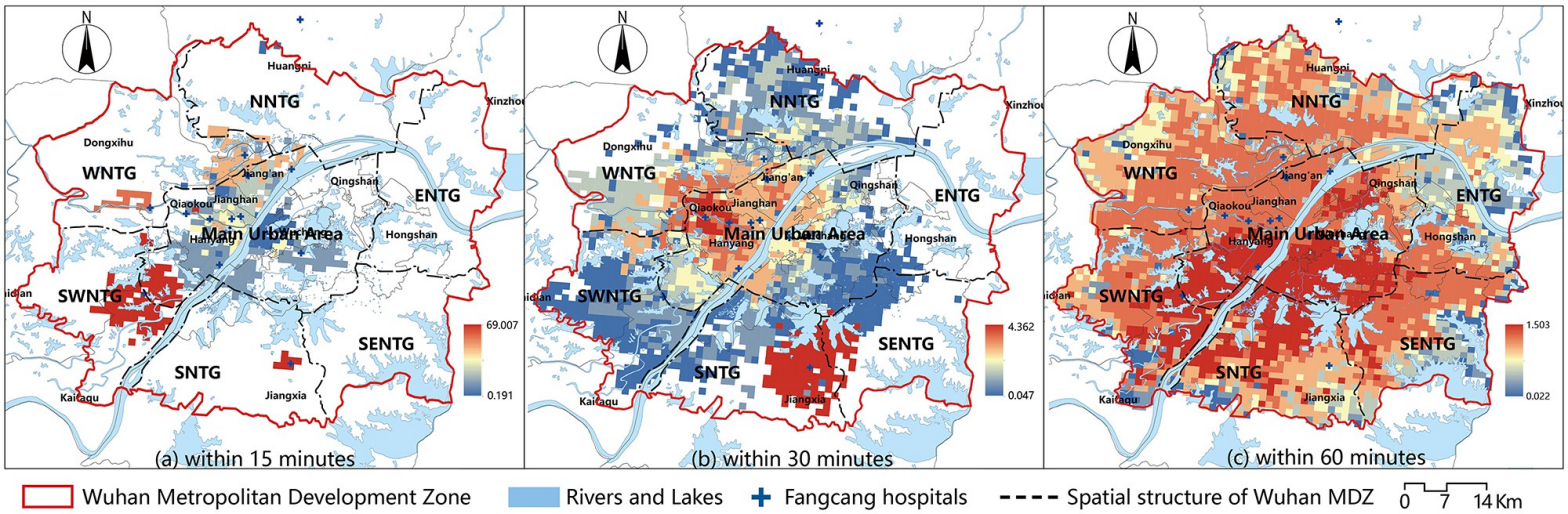
Accessibility of Fangcang hospitals. (a)Within 15 minutes, (b) Within 30 minutes, (c) Within 60 minutes.

Similarly, the accessibilities of demand points within 15, 30, and 60 mins of Fangcang hospitals was calculated using formula four. The value was then visualized in ArcGIS 10.6 using Jenks. Fig 7 depicts the outcomes. The data revealed that the Fangcang hospitals’ 15-minute service area was tiny, with 435 demand points able to reach the hospitals within 15 mins, accounting for 13.17% of the total. The regions with the best accessibility were near the Zhuankou Fangcang Hospital in the Southwest New Town Group (Fig 7a). More than half (52.92%) of the demand points could reach Fangcang hospitals in 30 mins. Most places in the Eastern New Town Group and Southeast New Town Group could not get to Fangcang hospitals within 30 mins, whereas areas with the highest accessibility were near the Jiangxia Fangcang Hospital (Fig 7b). Only 138 demand points that could not reach Fangcang hospitals when the time limit was set to 60 mins. These demand points were mostly found around the Wuhan MDZ’s easter border. The southern part of the main urban area, the northern part of the Southern New Town Group, and the northeast of the Southwest New Town Group had the best accessibility (Fig 7c).

**Fig 8:**
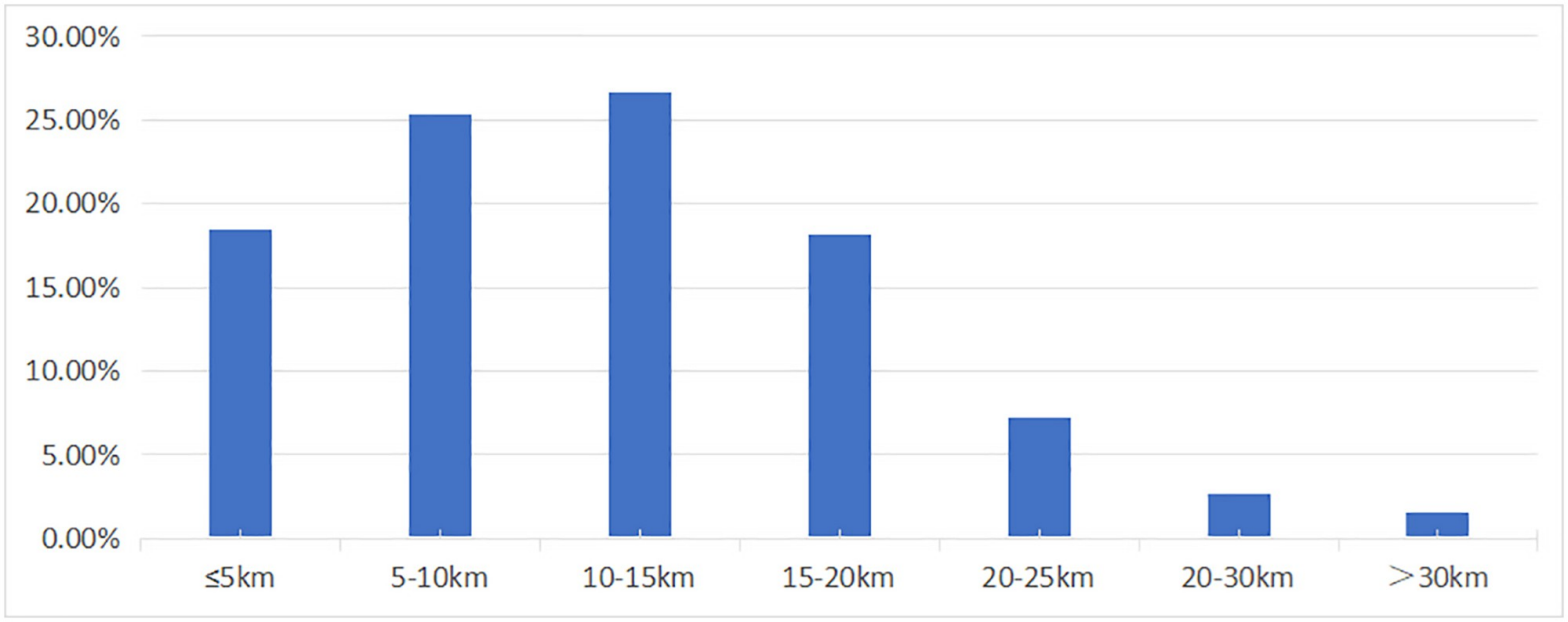
Distribution of distance cost of fever clinics for demand points.

**Fig 9:**
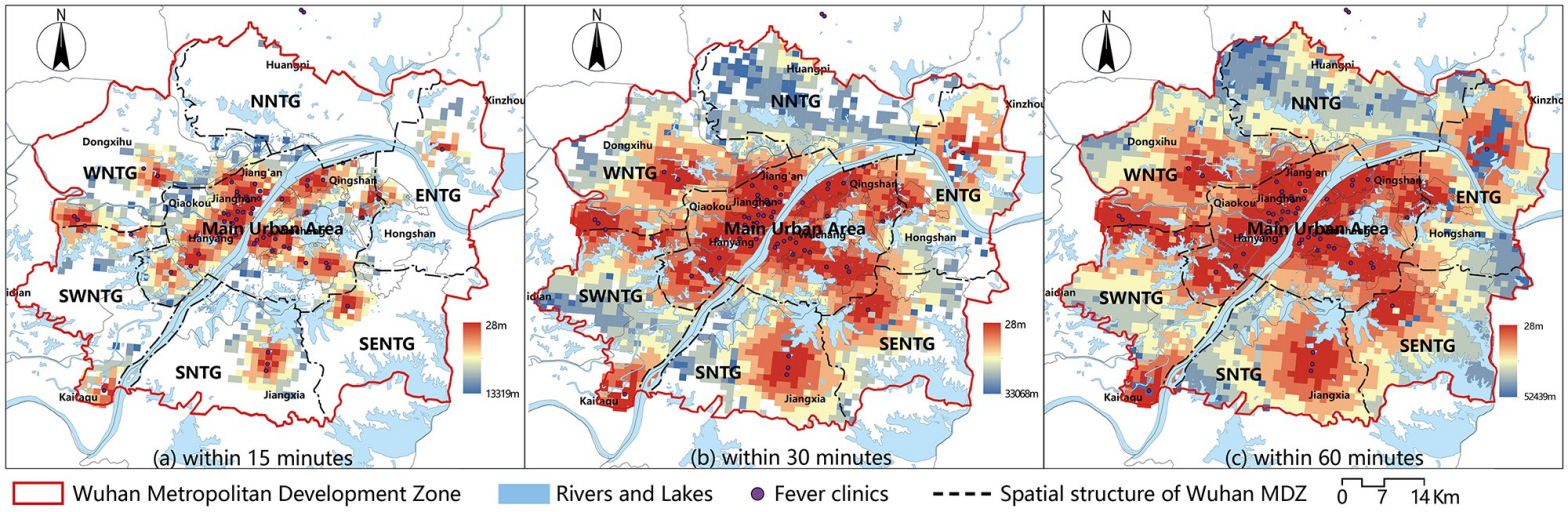
Shortest distance cost of fever clinics. (a)Within 15 minutes, (b) Within 30 minutes, (c) Within 60 minutes.

The distance cost of fever clinics for demand points was calculated using the shortest distance method, and the results were visualized (Figs 8 and 9). Fig 8 depicts how the distance between demand points to fever clinics in the Wuhan MDZ grew and then fell, displaying a substantial normal distribution with a peak in the range of 10–15 km, and accounting for 26.64% of the total. The proportion of distances in the range of 5–10 km was also significant, accounting for 25.34%. Demand points in the 5 km and 15–20 km ranges were similar in number, accounting for 18.47% and 18.17%, respectively. There were fewer demand points with a distance higher than 20 km (4.17%), with 52 exceeding 30 km accounting for 1.57%. The shortest distance between demand points and fever clinics was 11.74 km on average.

Fig 9 depicts the geographic variation in the accessibility of fever clinics. There were 927 demand points that could cover the needs of fever clinics within 15 mins, accounting for 28.06% (Fig 9a). The percentages of demand points that could reach fever clinics in 30 and 60 mins accounted for 77.08% and 99.54%, respectively. There were only a few demand points that could not get to fever clinics in under an hour (Fig 9b and 9c). Overall, the accessibility distribution showed a tendency of gradual deterioration from central to the periphery, with the main urban area having the best accessibility and the Northern New Town Group having the poorest.

### 4.2 Equality evaluation results based on the Gini coefficients

Based on the aforementioned accessibility data, Formula 5 was used to calculate the Gini coefficients of these medical facilities at various time thresholds. The maximum Gini coefficient is "1" and the minimum is "0", with the closer a value is to 0, the more equal distribution the resource are. In international practice, 0.2 is considered an absolute average, 0.2–0.3 a relative average, 0.3–0.4 a reasonable range, and 0.4–0.5 a large gap. When the Gini coefficient value approaches 0.5 or above, the resource distribution disparity is significant.

**Fig 10:**
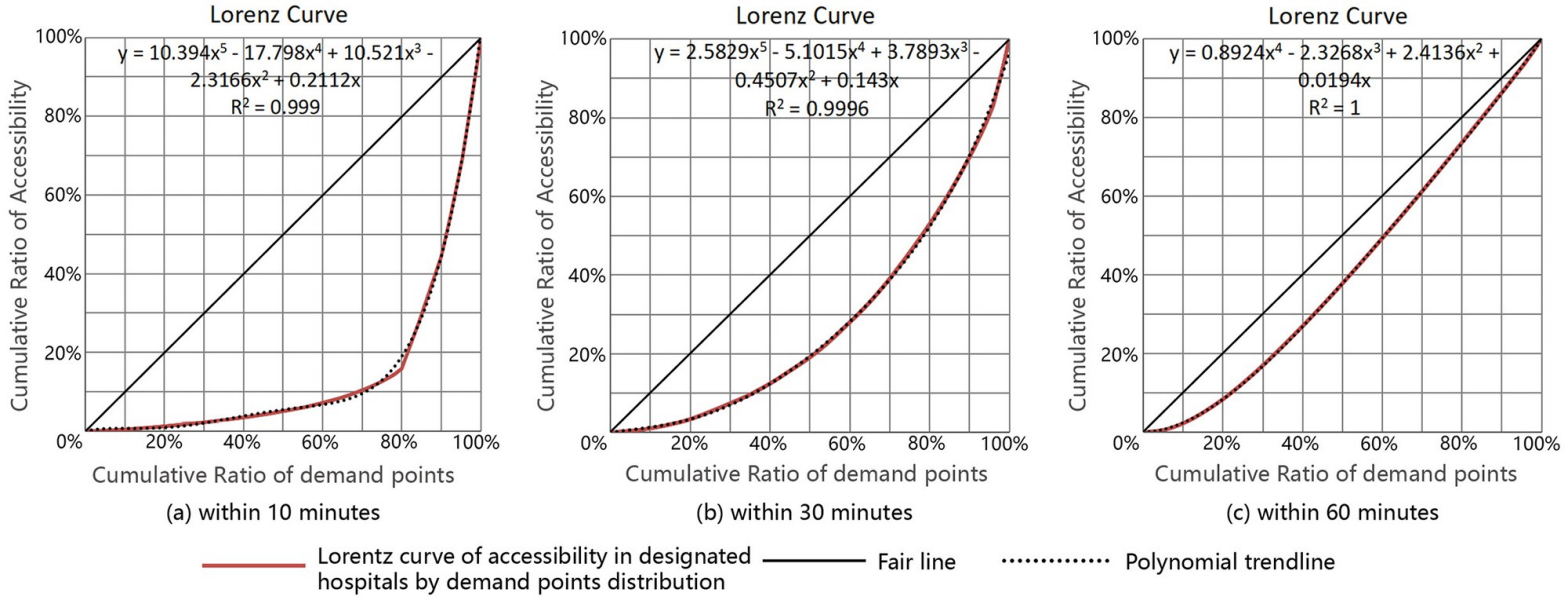
Lorentz curve of accessibility to designated hospitals by demand point distribution. (a)Within 10 minutes, (b) Within 30 minutes, (c) Within 60 minutes.

The Gini coefficients for the accessibility of designated hospitals at 10, 30, and 60 mins were 0.72, 0.44, and 0.17, respectively, according to the findings. It means that within 10 mins the equality of designated hospitals was inadequate. Furthermore, while equality within 30 mins was better than that within 10 mins, the disparity in several areas remained significant. When the time limit was set at one hour, equality was excellent, and accessibility in all areas was distributed equally. The Lorentz curve was drawn, as seen in Fig 10.

**Fig 11:**
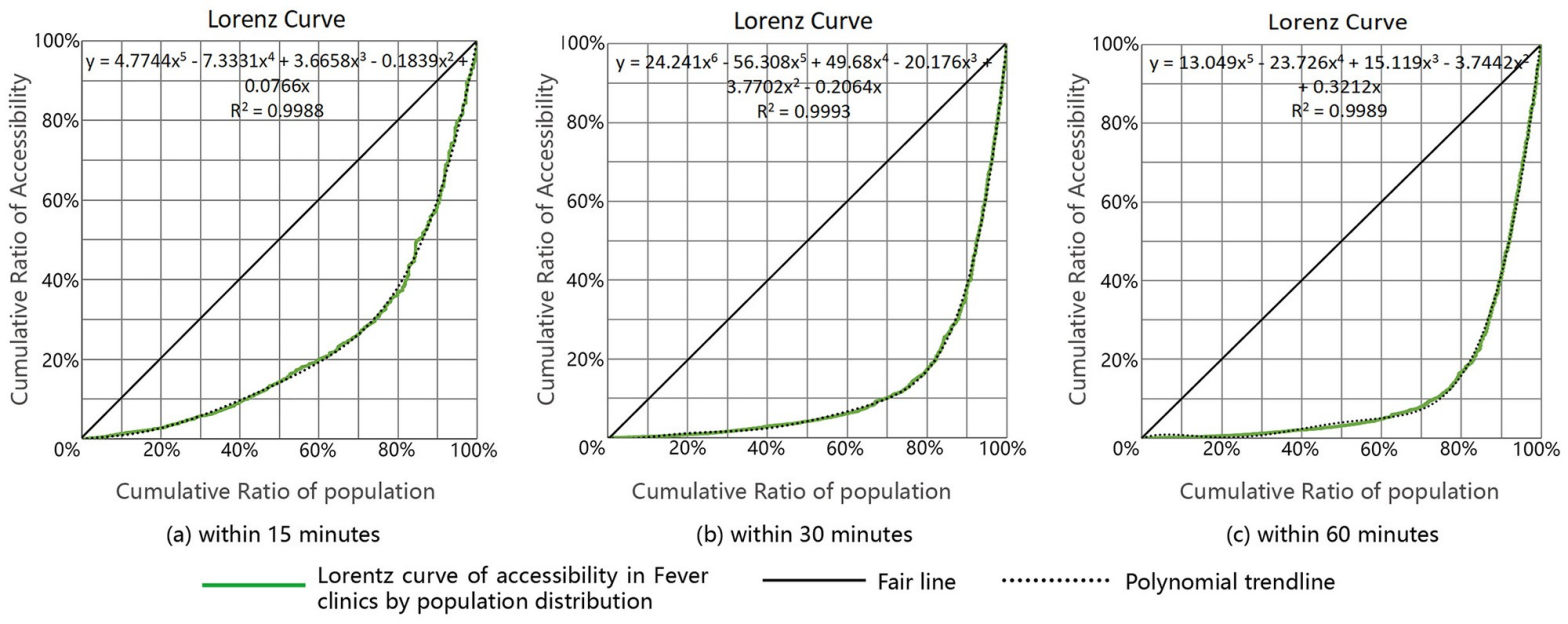
Lorentz curves of accessibility to fever clinics by population distribution. (a)Within 15 minutes, (b) Within 30 minutes, (c) Within 60 minutes.

The Gini coefficients for the accessibility of fever clinics at 15, 30, and 60 mins were 0.28, 0.31, and 0.32, respectively. However, as the shortest distance method was different from KD2SFCA, the results of accessibility did not reflect the relationship with the population of demand points; hence, we incorporated the population of demand points to calculate the Gini coefficient and draw the Lorentz curve (Fig 11). This curve reflects the cumulative ratio of demand point population and the cumulative ratio of the shortest distance cost. The Gini coefficients for 15, 30, and 60 mins were 0.55, 0.75, and 0.76, respectively, according to the findings.

## 5. Discussion

### 5.1 Major findings

The following are some of the study’s major findings:

The accessibility of pandemic treatment facilities in the Wuhan MDZ declined from the center to the periphery, with significant variance from one place to another. This matched Wuhan’s accessibility features for older medical facilities [23]. The main urban area had the best accessibility, whereas the Southeast and Eastern New Town Groups had the poorest. It may be due to urbanization. Medical facilities in main urban areas were fully equipped, allowing them to transition into designated hospitals and Fangcang hospitals in an outbreak. However, New Town Groups’ outdated infrastructure makes changes difficult.

The pandemic treatment facilities in the Wuhan MDZ need to be enhanced in terms of distance. More than 70% of demand points could not access these facilities within 15 minutes, whether they were designated hospitals or fever clinics. A lack of pandemic treatment and screening facilities caused this situation. While numerous medical facilities existed [50], they were not designed for pandemic treatment and could not be used in a pandemic.

The distribution of emergency treatment facilities during a pandemic was imbalanced. This matched the findings of previous research [51]. When the time limit was short (e.g., 10–15 min), the equality gap between various areas was larger. It means that demand points have better access to designated hospitals and Fangcang hospitals in non-emergency situations. Most demand points could reach hospitals within the “Golden Hour,” and spatial distribution equality was reasonable. However, accessibility was limited for severely sick patients who needed to arrive at the facilities and begin treatment within “Platinum Ten,” and the equality varied greatly. This was largely due to the concentration of pandemic treatment facilities in the main urban area.

Eastern Wuhan MDZ had the least access to Fangcang hospitals. There were two probable explanations. First, the pandemic influenced the location selection of Fangcang hospitals (i.e., as shown in Fig 2, the pandemic in the eastern region was not serious). Second, there were no public facilities suitable for Fangcang hospitals.

The Gini coefficient of fever clinics evaluated by the shortest distance was much lower than that calculated after the population was taken into account. It shows that the distribution of fever clinics in the study area was somewhat balanced in terms of distance. However, when the population was taken into account, the inequality gap became enormous.

### 5.2 Suggestions on spatial layout optimization

Based on the above results and findings, the following suggestions for improving the spatial distribution of pandemic treatment medical facilities in Wuhan are made:

Fangcang hospitals, as well as Leishenshan and Huoshenshan hospitals, play a crucial role in enhancing accessibility in the area during a pandemic as temporary emergency facilities. In order to ensure accessibility and equality of medical treatment after they close, it is required to replenish the number of beds in competent hospitals in the appropriate locations. Also, in order to eliminate the need for temporary hospitals in the future, designated hospitals must plan ahead of time for hospital expansion in the event of a pandemic.

To improve equality, the New Town Groups must develop pandemic treatment facilities. Despite each New Town Group having many medical facilities, the pandemic treatment facilities remained inadequate. Decision-makers should choose facilities with superior basic conditions for providing vital assistance, upgrade their hardware, and convert them into designated hospitals. Authorities should also encourage high-level hospitals in the main urban area to open branches in New Town Groups, improving pandemic treatment assurance. In addition, planners must avoid clustering hospitals of the same type and unevenly distributing them.

Each New Town Group should increase the number of fever clinics to promote short-time accessibility. However, the number of fever clinics in the main urban area cannot be increased. Instead, authorities can enlarge the building area of facilities and increase the number of doctors and nurses according to population density to meet the medical needs of adjacent inhabitants.

**Fig 12:**
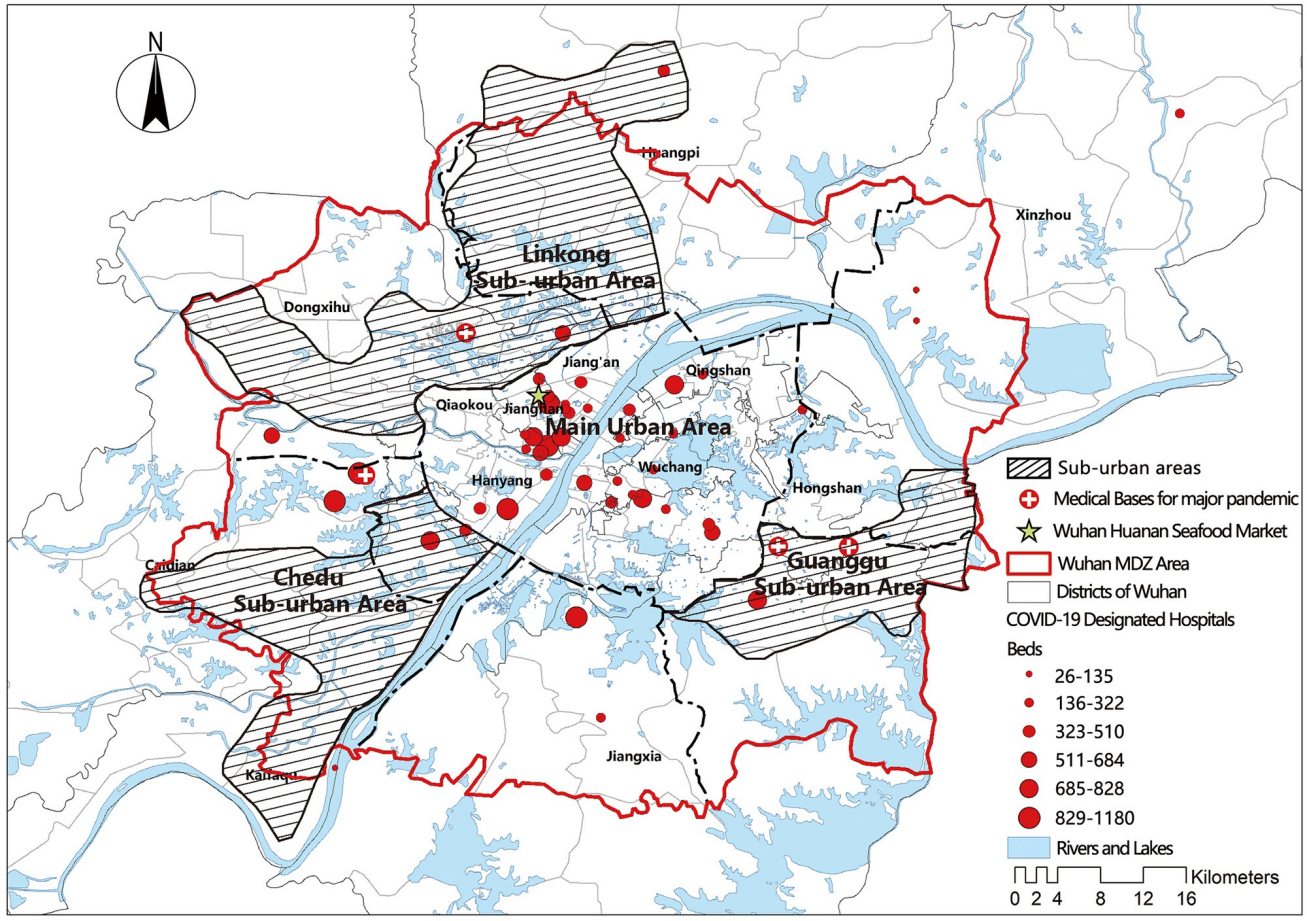
Distribution of sub-urban areas and existing designated hospitals in Wuhan.

According to the master plan of Wuhan City (2017–2035), except for the main urban area, three sub-urban areas, Guanggu, Chedu, and Linkong, will be created in the future. Because of the rising population and building density, pandemic treatment facilities, particularly designated hospitals, will need to be strengthened (Fig 12).

### 5.3 Strengths and limitations

This paper investigated the spatial distribution of medical facilities for pandemic treatment in Wuhan and made recommendations for improving the facilities’ spatial structure. It not only adds the research on the equal access to public service, but it also has an implication for the treatment of urban infectious diseases. This study used population data with a precision of 1km X 1km. Aside from being more accurate, the data is better equipped to cover the entire location of demand points. In addition, travel distance and time are real-time data based on network map navigation, with real-time traffic conditions such as speed restrictions and traffic light conditions included in. As a result, the findings of this study were more realistic. Furthermore, various accessibility calculation methods for different types of hospitals were utilized, as well as different travel time limitations. It suited better with the character and function of hospitals themselves than the one-size-fits-all approach. As a result, the study’s findings will be more relevant.

This study had several limitations. First, because the population data used in this study is ten years old, some errors were predicted in the results. Second, due to the large amount of data, travel time and distance were only collected on one day. Different days and times would produce different outcomes. Third, non-peak hours were chosen to simulate traffic conditions. In fact, off-peak traffic volumes are different from the scarcity of vehicles during a severe pandemic. Finally, residents’ access to medical treatment was linked to personal traits and travel preferences. However, this paper did not analyze these factors. Future polls could capture more accurate medical treatment preferences.

## 6. Conclusions

The accessibility calculation and equality evaluation of medical facilities play an important role in the spatial distribution optimization of facilities. Furthermore, optimizing the spatial distribution of pandemic treatment facilities is a crucial guarantee for pandemic prevention, control, and treatment. Using the KD2SFCA and the shortest distance methods, the accessibility of designated hospitals, Fangcang hospitals, and fever clinics in the Wuhan MDZ was evaluated using network map navigation data and population data with a 1 km^2^ precision. The Gini coefficient and Lorentz curve were then used to assess the equality of accessibility distribution. The results showed that most of the demand points in the study area can reach medical facilities for COVID-19 pandemic treatment within 60 minutes. However, because some demand points are inaccessible within a short time frame, emergency medical facilities for a pandemic need to be improved to better serve the population. Based on the results, several suggestions for facility spatial distribution optimization were made, which will be critical in the future improvement of Wuhan’s pandemic treatment medical facility network.

The next step was to optimize the spatial layout of the city’s pandemic treatment facilities and build a strong hierarchical pandemic treatment facility network by combining spatial layout optimization models (e.g., minimize resistance and maximize coverage model) with the division of land use in Wuhan’s master plan.
